# Identification of MicroRNA-92a-3p as an Essential Regulator of Tubular Epithelial Cell Pyroptosis by Targeting Nrf1 via HO-1

**DOI:** 10.3389/fgene.2020.616947

**Published:** 2021-01-11

**Authors:** Renhe Wang, Haijun Zhao, Yingyu Zhang, Hai Zhu, Qiuju Su, Haiyan Qi, Jun Deng, Chengcheng Xiao

**Affiliations:** ^1^Department of Traditional Chinese Medicine, Qingdao Municipal Hospital, Qingdao University, Qingdao, China; ^2^Department of Urology, Qingdao Municipal Hospital, Qingdao University, Qingdao, China

**Keywords:** renal ischemia–reperfusion injury, miR-92a-3p, Nrf1, pyroptosis, tubular epithelial cell, HO-1

## Abstract

Renal ischemia–reperfusion injury (IRI) is a major cause of acute kidney injury (AKI) and has no effective treatment. Exploring the molecular mechanisms of renal IRI is critical for the prevention of AKI and its evolution to chronic kidney disease and end-stage renal disease. The aim of the present study was to determine the biological function and molecular mechanism of action of miR-92a-3p in tubular epithelial cell (TEC) pyroptosis. We investigated the relationship between nuclear factor-erythroid 2-related factor 1 (Nrf1) and TEC pyroptosis induced by ischemia–reperfusion *in vivo* and oxygen–glucose deprivation/reoxygenation (OGD/R) *in vitro*. MicroRNAs (miRNAs) are regulators of gene expression and play a role in the progression of renal IRI. Nrf1 was confirmed as a potential target for miRNA miR-92a-3p. In addition, the inhibition of miR-92a-3p alleviated oxidative stress *in vitro* and decreased the expression levels of NLRP3, caspase-1, GSDMD-N, IL-1β, and IL-18 *in vitro* and *in vivo*. Moreover, Zn-protoporphyrin-IX, an inhibitor of heme oxygenase-1, reduced the protective effect of Nrf1 overexpression on OGD/R-induced TEC oxidative stress and pyroptosis. The results of this study suggest that the inhibition of miR-92a-3p can alleviate TEC oxidative stress and pyroptosis by targeting Nrf1 in renal IRI.

## Introduction

Acute kidney injury (AKI), a common clinical complication, presents with a sudden and persistent drop in renal function (Bellomo et al., 2012). The morbidity and mortality rates of AKI were reported to reach 5% and 50%–80%, respectively. This causes huge societal and personal economic burdens (Gill et al., 2005; Perico and Remuzzi, 2015). Surviving patients with AKI have a greater probability of contracting chronic kidney disease (CKD) and end-stage renal disease (ESRD) (Chawla et al., 2014), which reduces the recovery rate and worsens prognosis. Renal ischemia–reperfusion injury (IRI), which leads to shock and sepsis and requires kidney transplantation, is one of the leading causes of AKI (Gill et al., 2005; Bastin et al., 2013; Noh et al., 2015). Due to their high metabolic activity and hypoxia, renal tubular epithelial cells (TECs) are most influenced by renal IR (Al-Bataineh et al., 2016). Research indicates that TEC intervention effectively protects against renal IRI in terms of autophagy, apoptosis, and inflammation (Lin, 2017; Han and Lee, 2019). However, TEC pyroptosis in renal IRI is poorly understood.

The Nomenclature Committee on Cell Death defined pyroptosis as a pattern of regulated cell death mainly dependent on the formation of plasma membrane pores mediated by gasdermin proteins, which were activated by intra- and extracellular stimuli (Miao et al., 2010; Galluzzi et al., 2018). TEC pyroptosis is one of the main pathological features of renal IRI. Previous studies have shown that oxidative stress could trigger tubular injury and induce TEC pyroptosis, which in turn initiates oxidative stress. These two mechanisms were positively correlated with both mutual coordination and jointly aggravate kidney dysfunction (Diao et al., 2019; Tajima et al., 2019; Liu et al., 2020). Nuclear factor-erythroid 2-related factor 1 (Nrf1), a CNC-bZIP protein, is a key regulator of oxidative stress (Hou et al., 2018). It is an indicator of mitochondrial oxidative metabolism in renal diseases (Jiang et al., 2019), and its activation could prevent renal fibrosis (Huang et al., 2019). However, the role of Nrf1 in TEC pyroptosis is unclear.

MicroRNAs (miRNAs) are non-coding endogenous RNAs that inhibit translation by targeting messenger RNA (mRNA) (Ha and Kim, 2014). Research suggests that miRNAs are key regulators and potential biomarkers or therapeutic targets of renal IRI. These studies have also shown that miRNAs are crucial in renal IRI by regulating TEC pyroptosis (Wu et al., 2016; Song et al., 2019; Zhu et al., 2020). Thus, exploring the potential mechanism of miRNAs in renal IRI can be effective in preventing AKI and its transformation into CKD and ESRD. In this study, we explored whether the loss of miR-92a-3p prevents TECs from oxidative stress and pyroptosis by targeting Nrf1 via heme oxygenase-1 (HO-1).

Together, our current work provides insight into the mechanism by which miR-92a-3p regulates the pyroptosis of TEC in renal IRI.

## Materials and Methods

### Animals and Surgical Protocols

The guidelines provided in the Guide for the Care and Use of Laboratory Animals (1996) were followed during surgical procedures. Approval for the study was granted by the Institutional Animal Care and Use Committee of Qingdao Municipal Hospital. Beijing Huafukang Bioscience Co., Inc. provided C57BL/6 8-week-old male mice weighing 20–25 g. The mice were anesthetized by administering 2% sodium phenobarbital, weighing 50 mg/kg, into the peritoneum, and the loss of righting reflex was used to assess the correct depth of surgical anesthesia. The mice were then set on a homeothermic table to keep the rectal temperature at approximately 38°C. After completely exposing the bilateral pedicle, pedicle clamping was performed to induce ischemia. After 30 min, the clips were opened and the kidneys were collected at intervals of 6, 12, and 24 h of reperfusion. No pedicle clamping was performed during the same operation for the sham animals. The mice in the I/R model group and the sham group were then intravenously (via tail vein) injected with 10 mg/kg antagomir miR-92a-3p or antagomir (negative control, NC) (GenePharma Co., Ltd, Shanghai, China). A higher dose (200 mg/kg) of pentobarbital sodium was administered after the experiment to sacrifice the mice. Liquid nitrogen was used to snap-freeze the cutout left sided kidneys, and these were either preserved at −80°C or in 4% paraformaldehyde for biochemical analysis and pathological evaluation.

### Cell Culture and Transfection

The American Type Culture Collection provided TCMK-1 cells (no. CCL-139) for the study. A complete medium with 5% CO_2_ and 95% air was used to culture cells (an overall 1.5 × 10^6^ cells/ml) for comparison of the groups. Glucose-free medium was used to stimulate cells in OGD/R groups for 24 h under hypoxic conditions. Then, normal conditions were applied for 2, 4, and 8 h to culture cells in complete medium. Normoxic conditions were applied for culturing the control group. Lipofectamine 2000 (Invitrogen, Shanghai, China) was used to transfect antagomir miR-92a-3p or antagomir NC into TCMK-1. 0.25 M NaOH was added to Zinc protoporphyrin IX (ZnPPIX) (Wako Pure Chemical Industries, Osaka, Japan) to dilute the concentration to 10 μg/μl. TCMK-1 cells were then incubated with ZnPPIX for 24 h.

### Establishment of Nrf1-Overexpression Cell Line

Methods for establishing a cell line stably overexpressing Nrf1 protein have been described previously (Xiao et al., 2017, 2020). Briefly, EcoRI and SpeI (NEB) were used to digest pUC57-Nrf1 and pLVX-mCMV-ZsGreen-IRES-Puro (Wuhan Viral Therapy Technologies Co., Ltd). Next, T4 DNA Ligase by BM121 (TransGen BioTech) was used to connect the products, and fragments were recovered using an Agarose Gel Extraction Kit (Omega). Moreover, JM109 (Wuhan Viral Therapy Technologies Co., Ltd), possessing the ligation product after transfection, underwent inoculation and amplification using the sequencing primer CMV-F (5′-CGCAAATGGGCGGTAGGCGTG-3′) to corroborate the bacterial fluid. Lentiviral packaging kit (Wuhan Viral Therapy Technologies Co., Ltd) was used to obtain rLV-Nrf1 and rLV-ShRNA2 possessing the gene to be targeted by co-transfecting 293T cells with either recombinant or control plasmid. Transfection of TCMK-1 cells with rLV-Nrf1 and rLV-ShRNA2 was carried out based on MOI = 20. TCMK-1 cells transfected with lentivirus were cultured for 2 days after transfection.

### TUNEL Assay

The staining of 4-μm-thick sections was performed using a TUNEL kit (cat no. 11684817910, Roche), following the manufacturer’s instructions. Nuclei of cells with brown staining were described as TUNEL-positive. Five images were randomly selected under a microscope (×400 magnification).

### Cell Counting Kit-8 (CCK-8) Assay

Cell viability was measured using the CCK-8 assay kit (cat no. C0038, Beyotime) according to the manufacturer’s instructions. Absorbance was measured at 450 nm using a microplate reader (Molecular Devices).

### Lactate Dehydrogenase (LDH) Release Assays

Cells were inoculated in 96-well plates and culture supernatants were collected. An LDH release assay kit (cat no. C0017, Beyotime) was used to assess the degree of LDH release following the manufacturer’s instructions. Absorbance was measured at 495 nm using a microplate reader.

### Dual-Luciferase Reporter Assay

The wild-type and mutant Nrf1-3′UTR constructs were cloned into the pGL3 vector, giving us wt-pGL3-Nrf1-3′UTR and mut-pGL3-Nrf1-3′UTR. The reporter plasmid was transfected into 293T cells kept in 24-well plates using Lipofectamine 2000. After 48 h, we detected and analyzed the activity of luciferase.

### Reverse Transcription-Quantitative PCR (RT-qPCR)

We isolated the entire RNA of both renal samples and TCMK-1 cells using TRIzol reagent (Invitrogen). For quantification and reverse transcription of extracted RNA, the Revert Aid First Strand cDNA Synthesis kit (cat no. K1622, Invitrogen) was used, following the manufacturer’s instructions. Real-time quantitative PCR for β-actin and miR-92a-3p was performed using the following primers: Nrf1, forward 5′-TTACTCTGCTGTGGCTGATGG-3′, reverse 5′-CCTCTGATG CTTGCGTGGTCT-3′; GSDMD-N, forward 5′-GTGTGTCAA CCTGTCTATCAAGG-3′, reverse 5′-CATGGCATCGTAGAAG TGGAAG-3′; β-actin, forward 5′-TACCTGAAGCCCCAAC TACAAA-3′, reverse 5′-GTGCCCTGCCACATGATAAA-3′; U6 forward 5′-GCTTCGGCAGCACATATACTAAAAT-3′, reverse 5′-CGCTTCACGAATTTGCGTGTCAT-3′; miR-92a-3p, forward 5′-TATTGCACTTGTCCCGGCCTG-3′, reverse 5′-TGTCGTGGAGTCGGCAATTG-3′. The relative expression of miR-92a-3p was normalized with U6, and the relative expression of Nrf1 and GSDMD-N was normalized with β-actin. We adopted the 2^–((^*^*Cq*^* method to ascertain the comparative expression (Livak and Schmittgen, 2001).

### Enzyme-Linked Immunosorbent Assay

For detection of the degree of expression of interleukin (IL)-1β and IL-18 within the medium, the IL-1β and IL-18 ELISA kits (IL-1β, cat no. ab197742; IL-18, cat no. ab216165, Abcam) were used as recommended by the manufacturer. A microplate reader was used to measure absorbance at 495 nm, while a plate reader was used to read the ELISA signal.

### Hematoxylin and Eosin Staining

Kidney tissues were fixed in 4% paraformaldehyde. These tissues were sliced into 4-μm-thick pieces while embedded in paraffin. Hematoxylin and eosin kit (cat no. C0105M, Beyotime) was then used to stain sections of tissues following the manufacturer’s instructions. For analyzing pathological alterations, a microscope (×400 magnification) was used to take five random images. The percentage of tubules that were affected was taken as tubule injury scores as follows: 0 = ≤10%, 1 = 10%–25%, 2 = 26%–50%, 3 = 51%–75%, and 4 = ≥75%.

### ROS, MDA, and SOD Measurements

To obtain supernatant, after cells were transfected and OGD/R induction was carried out, cells were centrifuged at room temperature for 10 min at 4000 (*g*. The quantities of reactive oxygen species (ROS, cat no. S0033M, Beyotime), malondialdehyde (MDA, cat no. S0131M, Beyotime), and superoxide dismutase (SOD, cat no. S0109, Beyotime) were detected following the manufacturer’s instructions.

### BUN and Serum Creatinine Levels Assay

BUN and creatinine levels were measured using commercially available assay kits (Urea Nitrogen B Test for BUN and LabAssay^TM^ Creatinine for creatinine, Wako, Osaka).

### Flow Cytometric Analysis

Stimulated cells were stained first with annexin V and then with propidium iodide using an Annexin V-FITC Apoptosis Detection kit (cat no. 558064, BD Biosciences). Flow cytometry was then used to analyze apoptotic cells.

### Immunohistochemistry (IHC)

Hydrogen peroxide (3%) was used to block 5-μm-thick sections after deparaffinizing and hydration. The sections were then treated with 10% normal goat serum (cat no. 16210072, Gibco, Thermo Fisher Scientific, Inc.) and incubated overnight after adding antibodies targeting IL-1β (1:100, cat no. ab9722, Abcam), IL-18 (1:200, cat no. Ab71495, Abcam), GSDMD-N (cat no. bs-14287R, Bioss), and Nrf1 (cat no. ab175932, Abcam). Afterward, sections were incubated with horseradish peroxidase (HRP)-conjugated goat anti-rabbit IgG secondary antibodies (Thermo Fisher Scientific, Inc.). 3,3′-Diaminobenzidine (DAB) was used to stain the slides to develop color and a microscope (×400 or ×200 magnifying power) was used to capture the images.

### Immunofluorescence Staining

TCMK-1 cells were fixed with 4% paraformaldehyde, after which 0.1% Triton X-100 was used to create permeability and 10% donkey serum (cat no. D9663, Sigma-Aldrich) was used for blocking. The cells were incubated overnight with antibodies against Nrf1 (1:100; cat no. ab175932, Abcam). The cells were then incubated with secondary antibodies (1:400; cat no. A32731, Invitrogen). Nuclei were stained using DAPI (Roche). Fluorescent images were captured under a microscope at ×400 magnification.

### Western Blotting

The BCA assay kit (Invitrogen) was used to quantify proteins after extraction. Nrf1, GSDMD-N, nod-like receptor protein-3 (NLRP3), caspase-1, HO-1, and β-actin expression were measured. The following antibodies were used for incubating the membranes overnight: anti-GSDMD-N (1:100; cat no. bs-14287R, Bioss), β-actin (1:500; cat no. ab8224, Abcam), anti-HO-1 (1:200; cat no. ab68477, Abcam), anti-NLRP3 (1:200; cat no. ab214185, Abcam), anti-Nrf1 (1:100; cat no. ab175932, Abcam), and anti-caspase-1 (1:200; cat no. ab138483, Abcam). Subsequently, HRP-conjugated secondary antibodies (1:500; cat no. ab6789, Abcam) were used to incubate the membranes. Finally, the Odyssey Infrared Imaging system (LI-COR Biotechnology) was used to analyze and detect the protein bands.

### Statistical Analysis

We performed statistical analysis using SPSS version 22.0 software. Data have been presented as mean ± standard deviation (SD). Pearson’s correlation test was applied to test the linear correlations of variables. All experiments were done in triplicate. Variance analysis was used to evaluate statistical significance and *P* values < 0.05 were considered to demonstrate statistically significant differences.

## Results

### Nrf1 Is Involved in TEC Pyroptosis in Renal IR

**FIGURE 1:**
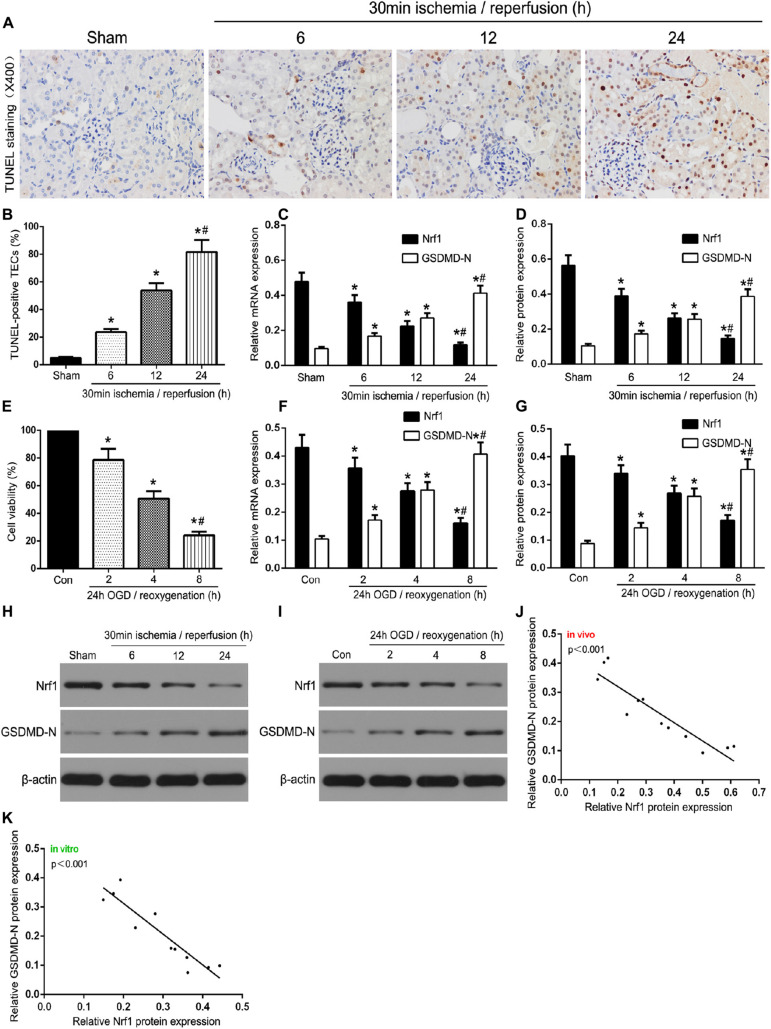
Nrf1 was involved in TEC pyroptosis in I/R-induced kidney and OGD/R-stimulated TCMK-1 cells. **(A)** Representative photomicrographs of tubular cell injury in mouse kidney tissue sections with TUNEL staining, 400×, scale bar = 20 μm. **(B)** Statistical analysis showed the percentage of TUNEL-positive TECs in the kidney tissues exposed to 30 min ischemia followed by reperfusion of different durations (6, 12, and 24 h). **(C)** Representative photomicrographs of Nrf1 and GSDMD-N expression in mouse kidney tissue sections by immunohistochemistry, 200×, scale bar = 20 μm. **(D)** qRT-PCR analysis of Nrf1 and GSDMD-N expression at different reperfusion times after 30 min ischemia in kidney samples. **(E,I)** Western blot analysis of Nrf1 and GSDMD-N expression at different reperfusion times after 30 min ischemia in kidney samples. **(F)** Cell viability was detected by CCK-8 assay. **(G)** qRT-PCR analysis of Nrf1 and GSDMD-N expression at different durations of reoxygenation after 24 h OGD in TCMK-1 cells. **(H,J)** Western blot analysis of Nrf1 and GSDMD-N expression at different durations of reoxygenation after 24 h OGD in TCMK-1 cells. **(K,L)** The correlation between the protein expression of Nrf1 and GSDMD-N was analyzed by Pearson’s correlation analysis in the kidney tissues and TCMK-1 cells. Data are expressed as the mean ± SD. *n* = 6 per group *in vivo* and *n* = 3 per group *in vitro*. *P < 0.05 vs. sham or con, #*P* < 0.05 vs. 30 min ischemia and reperfusion of 12 h *in vivo* or 24 h OGD and reoxygenation of 4 h *in vitro*, one-way ANOVA.

To investigate the role of Nrf1 in TEC pyroptosis, sampling was performed and the assay was carried out at 6, 12, or 24 h reperfusion after *in vivo* ischemia for 30 min, and then at 2, 4, or 8 h of re-oxygenation following 24 h of OGD *in vitro*. As demonstrated in Figures 1A,B, as the reperfusion time increased, the number of TUNEL-positive TECs also gradually increased. As shown in Figure 1C, with renal 30 min ischemia/24 h reperfusion, the expression of Nrf1 was decreased in TECs, and the GSDMD-N protein was accumulated in TEC membranes. Western blotting and qRT-PCR were used to evaluate Nrf1 and GSDMD-N expression in mouse samples. The results indicated a gradual decrease and increase in Nrf1 and GSDMD-N expression, respectively, upon reperfusion after ischemia for 30 min (Figures 1D,E,I). Assessment of TCMK-1 cells was carried out at 2, 4, or 8 h of re-oxygenation after *in vitro* OGD for 24 h. The CCK-8 assay (Figure 1F) demonstrated that OGD/R reduced the viability of TCMK-1 cells, and this gradually declined with increased duration of reoxygenation after 24 h of OGD. Consistent with the *in vivo* experiments, Nrf1 expression decreased and GSDMD-N expression increased steadily with prolonged intervals of reoxygenation after 24 h of OGD in TCMK-1 cells (Figures 1G,H,J). Moreover, the analysis of Pearson’s correlation revealed that Nrf1 and GSDMD-N expression had a negative correlation in the kidney induced by IR and OGD/R-stimulated TECs (Figures 1K,L). These results indicate that Nrf1 might be an important regulatory factor in TEC pyroptosis.

### Role of NRF1 in Pyroptosis in OGD/R-Stimulated TCMK-1 Cells

**FIGURE 2:**
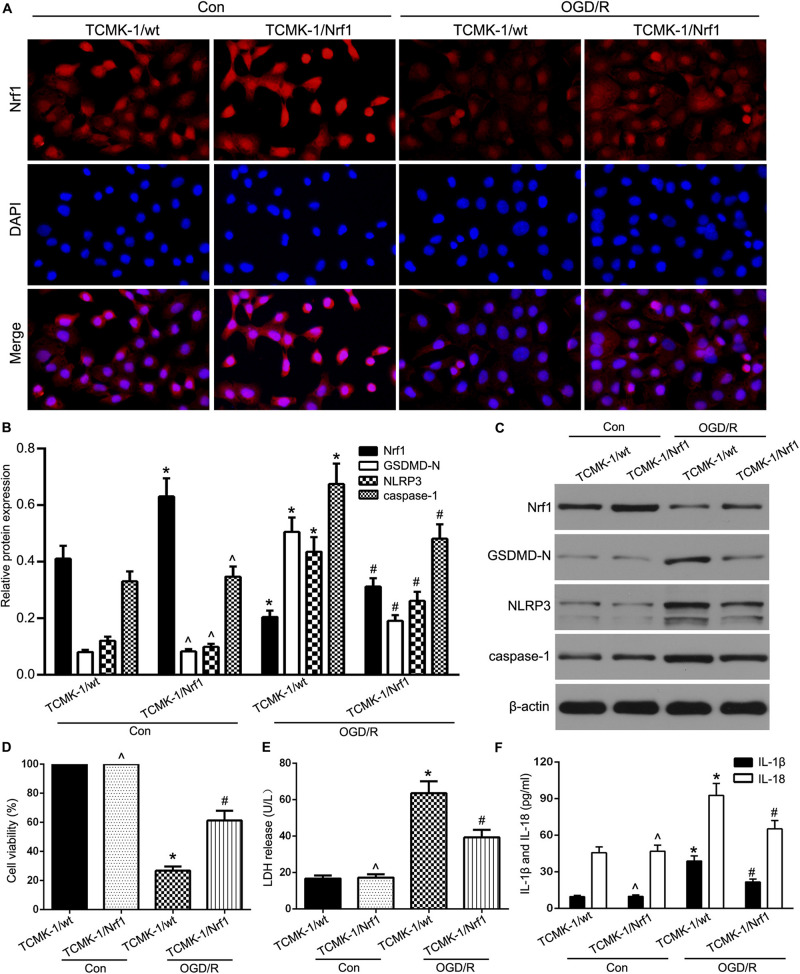
Nrf1 overexpression alleviated TEC pyroptosis in OGD/R-stimulated TCMK-1 cells. **(A)** Representative photomicrographs of Nrf1 expression in TCMK-1 cells by immunofluorescence, 400×, scale bar = 20 μm. **(B,C)** Western blot analysis of Nrf1, GSDMD-N, NLRP3, and caspase-1 expression in TCMK-1 cells. **(D)** Cell viability was detected by CCK-8 assay. **(E)** The LDH release was detected by activity assays. **(F)** IL-1β and IL-18 contents were measured by using ELISA kits. Data are expressed as the mean ± SD. *n* = 3 per group. *P < 0.05 vs. TCMK-1/wt con group, ∧*P* > 0.05 vs. TCMK-1/wt con group, #*P* < 0.05 vs. OGD/R-induced TCMK-1/wt group, one-way ANOVA.

To validate the role of Nrf1 in renal IRI, a lentivirus-based Nrf1 overexpression cell line (TCMK-1/Nrf1) or a vector-transfected cell line (TCMK-1/vector) was stimulated with 24 h of OGD and 8 h of reoxygenation. As shown in Figure 2A, the enhanced expression of Nrf1 in TCMK-1/Nrf1 cells with respect to TCMK-1/vector cells and Nrf1 expression decreased in OGD/R-stimulated TCMK-1 cells compared to the control groups. As shown in Figures 2B,C, Western blotting indicated that Nrf1 overexpression cell lines were successfully conducted, and the expression of NRF1 was abridged in TCMK-1 cells that were sparked by OGD/R. In addition, NLRP3, GSDMD-N, and caspase-1 expression were examined through Western blotting and showed that passing the cells through OGD/R resulted in a rise in the expression levels of GSDMD-N, NLRP3, and caspase-1 that had declined due to Nrf1 overexpression in comparison to the vector groups (Figures 2B,C). As shown in Figures 2D–F, Nrf1 overexpression expanded cell viability and reduced the level of LDH release, in addition to IL-1β and IL-18 expression. These results suggest that Nrf1 overexpression inhibits pyroptosis in TCMK-1 cells sparked by OGD/R.

### Nrf1 Is a Potential Target of miR-92a-3p

**FIGURE 3:**
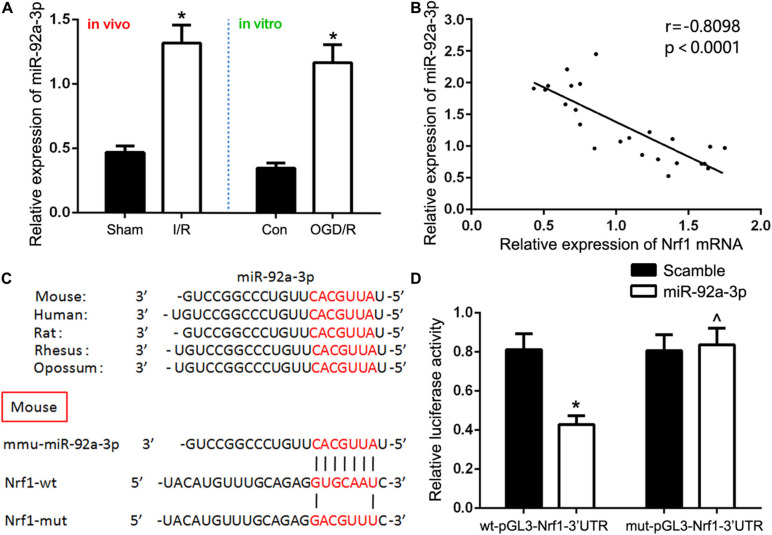
Nrf1 is a direct target of miR-92a-3p. **(A)** qRT-PCR analysis of miR-92a-3p expression *in vivo* and *in vitro*. **(B)** Pearson’s correlation analysis revealed that the mRNA expression of miR-92a-3p was inversely correlated with the expression of Nrf1. **(C)** Database predicted the existence of binding sites between Nrf1 3(UTR and miR-92a-3p. **(D)** Dual-reporter luciferase assay of miR-92a-3p expression in wt-pGL3-Nrf1-3(UTR or mut-pGL3-Nrf1-3(UTR constructs. Data are expressed as the mean ± SD. *n* = 6 per group *in vivo* and *n* = 3 per group *in vitro*. *P < 0.05 vs. sham, control, and wt-pGL3-Nrf1-3′UTR group. ∧*P* < 0.05 vs. mut-pGL3-Nrf1-3′UTR group, one-way ANOVA.

Bioinformatics analyses (TargetScan, miRDB, and Diana) were used to predict a binding site conserved for miR-92a-3p at the 3′UTR of Nrf1. To confirm this prediction, we used qRT-PCR to detect miR-92a-3p expression. We discovered that miR-92a-3p was significantly overexpressed in the renal IR group in comparison to the sham group *in vivo*, as well as in TCMK-1 cells sparked by the OGD/R, in comparison to the control group *in vitro* (Figure 3A). We also found that Nrf1 mRNA expression was negatively correlated with expression of miR-92a-3p in TCMK-1 cells (Figure 3B). In addition, 293T cells with dual-luciferase reporters were co-transfected with either wild-type (wt-pGL3-Nrf1-3′UTR) or mutant (mut-pGL3-Nrf1-3′UTR) plasmid as well as miR-92a-3p mimic or NC. The overexpression of miR-92a-3p evidently lowered the expression of Nrf1 in the wild type but not the mutant type (Figures 3C,D). Therefore, we hypothesized that miR-92a-3p might regulate Nrf1 expression in renal IRI.

### Inhibition of miR-92a-3p Alleviates Oxidative Stress and Pyroptosis in OGD/R-Induced TCMK-1 Cells

**FIGURE 4:**
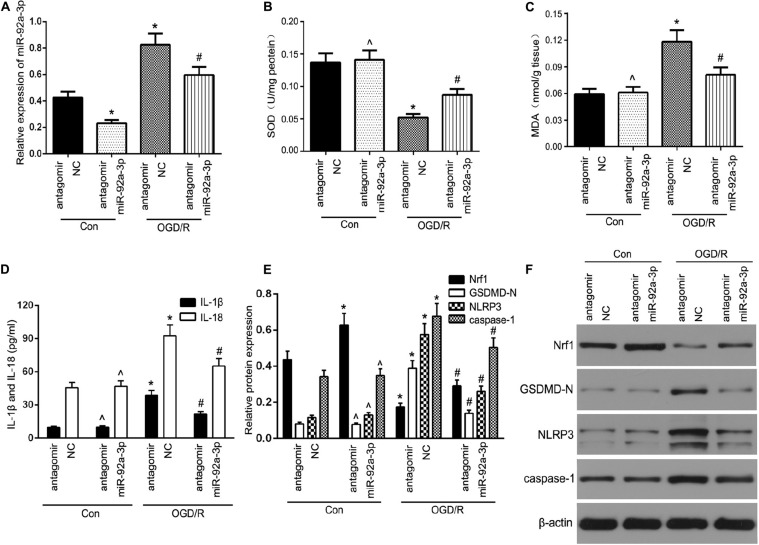
The inhibition of miR-92a-3p alleviated oxidative stress and pyroptosis in OGD/R-induced TCMK-1 cells. **(A)** qRT-PCR analysis of miR-92a-3p expression in TCMK-1 cells. **(B,C)** SOD and MDA levels were measured by using ELISA kits in TCMK-1 cells. **(D)** IL-1β and IL-18 contents were measured by using ELISA kits. **(E,F)** Western blot analysis of Nrf1, GSDMD-N, NLRP3, and caspase-1 expression in TCMK-1 cells. Data are expressed as the mean ± SD. *n* = 3 per group. *P < 0.05 vs. antagomir NC con group, ∧*P* < 0.05 vs. antagomir NC con group, #*P* < 0.05 vs. antagomir NC OGD/R group, one-way ANOVA.

To test this hypothesis, we first assessed miR-92a-3p expression by RT-qPCR. The results showed that transfection with antagomir miR-92a-3p dramatically reduced miR-92a-3p expression within TCMK-1 cells (Figure 4A). Oxidative stress-induced pyroptosis and its mediated-proinflammatory programmed cell death are important pathological processes in renal IRI (Diao et al., 2019; Liu et al., 2020). Given that Nrf1 is a key regulator of oxidative stress, we further investigated whether miR-92a-3p plays a regulatory role in oxidative stress in renal IRI. TCMK-1 cells passed through transfection with antagomir miR-92a-3p or antagomir NC were prompted by OGD/R. The results showed that the inhibition of miR-92a-3p profoundly increased SOD and decreased MDA in TCMK-1 cells that were sparked by OGD/R (Figures 4B,C). On the other hand, ELISA experiments showed that inhibition of miR-92a-3p considerably decreased the expression of IL-1β and IL-18 in TCMK-1 cells that had been induced by OGD/R, but not those under normal conditions (Figure 4D). In addition, we discovered that the downregulation of miR-92a-3p augmented the expression level of Nrf1 and lowered the levels of expression of NLRP3, GSDMD-N, and caspase-1 compared to the antagomir NC group induced by OGD/R (Figures 4E,F).

### Inhibition of miR-92a-3p Alleviates TEC Pyroptosis in the IR-Induced Kidney of Mice

**FIGURE 5:**
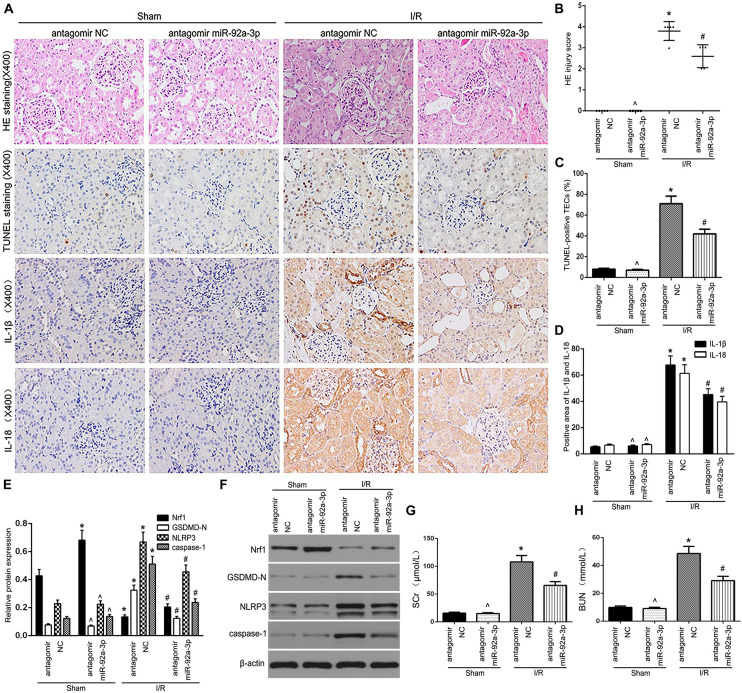
The inhibition of miR-92a-3p alleviated TEC pyroptosis in I/R-induced kidney of mice. **(A)** Representative photomicrographs of tubular cell injury in mouse kidney tissue sections with HE staining, TUNEL staining, and representative photomicrographs of IL-1β and IL-18 expression in mouse kidney tissue sections by immunohistochemistry, 400×, scale bar = 20 μm. **(B)** Statistical quantification analysis showed the injury score of HE staining in the kidney tissues. **(C)** Statistical analysis showed the percentage of TUNEL-positive TECs in the kidney tissues. **(D)** Statistical analysis showed the positive area of IL-1β and IL-18 in the kidney tissues. **(E,F)** Western blot analysis of Nrf1, GSDMD-N, NLRP3, and caspase-1 expression in mouse kidney tissue sections. SCr levels **(G)** and BUN levels **(H)** were detected in mice. Data are expressed as the mean ± SD. *n* = 5 per group. *P < 0.05 vs. antagomir NC sham group. ∧*P* > 0.05 vs. antagomir NC sham group. #*P* < 0.05 vs. I/R-induced antagomir NC group, one-way ANOVA.

To further assess the functional relevance of miR-92a-3p, kidney sampling was performed and assayed at 24 h following reperfusion after 30 min of ischemia. HE staining suggested that antagomir miR-92a-3p could not change renal structures without the treatment of IR. However, the induction of mice kidneys by IR demonstrated profoundly elevated renal tubular damage, which was markedly attenuated in the antagomir miR-92a-3p group (Figures 5A,B). Results showed that the number of TUNEL-positive TECs in IR-induced kidneys decreased, followed by miR-92a-3p inhibition (Figures 5A,C). IHC results showed that the downregulation of miR-92a-3p led to the decelerated expression of IL-1β and IL-18 already induced by IR, compared to the antagomir NC group (Figures 5A,D). In addition, when miR-92a-3p was inhibited, it enhanced the level of Nrf1 and decreased the levels of NLRP3, caspase-1, and GSDMD-N expression in IR-induced kidneys (Figures 5E,F). Meanwhile, the levels of both BUN and SCr were markedly increased in renal IR group and attenuated by miR-92a-3p inhibition (Figures 5G,H). Together, our results indicate that the inhibition of miR-92a-3p alleviates tubular pyroptosis in renal IRI by targeting Nrf1.

### MiR-92a-3p Regulates TCMK-1 Pyroptosis via HO-1 Targeting Nrf1

**FIGURE 6:**
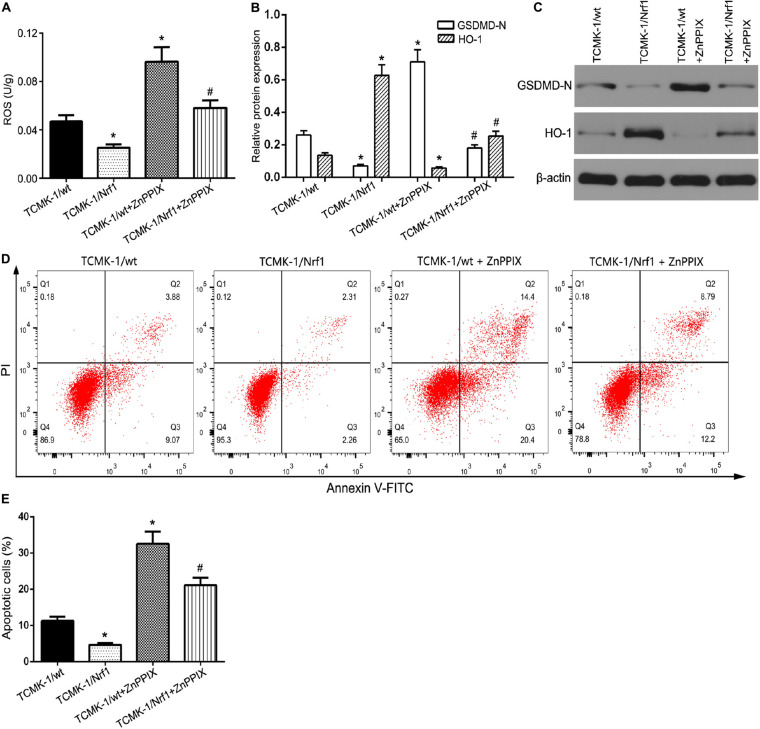
Nrf1 regulated TCMK-1 apoptosis via HO-1. **(A)** ROS levels were measured by using ELISA kits in TCMK-1 cells. **(B,C)** Western blot analysis of GSDMD-N and HO-1 expression in TCMK-1 cells. **(D)** Flow cytometry assays were performed to show the cell pyroptosis. **(E)** Statistical analysis was used to show pyroptosis cells. Data are expressed as the mean ± SD. *n* = 3 per group. *P < 0.05 vs. TCMK-1/wt group, #*P* < 0.05 vs. TCMK-1/wt + ZnPPIX group, one-way ANOVA.

Reactive oxygen species (ROS) and inflammatory responses affect the severity of renal IR injury (Gyuraszova et al., 2020). OGD/R-treated TCMK-1/Nrf1 cells or TCMK-1/vector cells were co-transfected with either antagomir miR-92a-3p or antagomir NC. As shown in Figure 6A, Nrf1 overexpression profoundly reduced the level of ROS in OGD/R-treated TCMK-1 cells, while ZnPPIX (a HO-1 inhibitor) partially blocked the protective effect of Nrf1 overexpression on ROS inhibition. In addition, the results of flow cytometry and Western blotting indicated that ZnPPIX reduced the effect of Nrf1 overexpression on the inhibition of pyroptosis and its related genes such as NLRP3, caspase-1, GSDMD-N, IL-1β, and IL-18 (Figures 6B–E). The above results show that miR-92a-3p regulates TCMK-1 pyroptosis by targeting Nrf1 via HO-1.

## Discussion

It is widely known that the kidneys are one of the organs most vulnerable to IRI (Ravikumar et al., 2016), in which TECs are involved in a series of complex molecular mechanisms, including oxidative stress, inflammatory response, intracellular calcium overload, and apoptosis (Linkermann et al., 2014). The role of TEC pyroptosis in renal diseases has attracted increasing attention. In this study, we found that Nrf1 was downregulated in IR-induced kidneys and TCMK-1 cells sparked by OGD/R and that the overexpression of Nrf1 was protected against TEC pyroptosis induced by OGD/R. It was discovered that miR-92a-3p could directly target Nrf1. Inhibition of miR-92a-3p exerted its protective effects against renal IRI by suppressing TEC oxidative stress and pyroptosis targeting Nrf1 via HO-1.

Nrf1, an important transmembrane transcription factor, is located in the endoplasmic reticulum and forms a heterodimer with small Maf or other bZIP proteins. The combination of the heterodimer with an antioxidant response element (ARE) regulates various physiological processes such as energy homeostasis, apoptosis, and inflammatory response (Evans and Scarpulla, 1990; Scarpulla, 1996; Zhang and Manning, 2015). Previous studies have shown that Nrf1 might act as a novel renal fibrosis antagonist in renal fibroblast cells (Hsieh et al., 2016). Therefore, we speculated that Nrf1 might be involved in the early stages of renal tubule damage. We found that Nrf1 was significantly decreased in IR-induced mouse kidneys as well as in OGD/R-stimulated TCMK-1 cells. In addition, Nrf1 expression decreased gradually with prolonged durations of reperfusion after 30 min of ischemia in kidney tissues and reoxygenation after 24 h of OGD in TCMK-1 cells. This suggests that Nrf1 is involved in early pathological processes in renal IRI. Next, we explored the mechanisms of Nrf1 in renal IRI. The characteristics of pyroptosis, which is a special form of programmed cell death, are completely different from apoptosis and necrosis due to excessive inflammation and cell death (Miao et al., 2010). GSDMD is mainly responsible for the execution of cell pyroptosis (Kovacs and Miao, 2017). Exposed to injury stimulation, GSDMD protein is cleaved into GSDMD-N and GSDMD-C by catalytic caspase-1. GSDMD-N produces pores in the cell membrane, leading to swelling and disruption of the cells with the release of inflammatory factors (Shi et al., 2015). In our study, the pyroptosis level of TECs was assessed by measuring the expression of GSDMD-N (Xiao et al., 2020), combined with TUNEL staining *in vivo* and cell viability assays *in vitro*. We found that TEC pyroptosis was aggravated gradually over time after renal IRI *in vivo* and *in vitro*. Moreover, the analysis of Pearson’s correlation revealed that Nrf1 and GSDMD-N expression had a negative correlation with the kidney induced by IR and OGD/R-stimulated TECs. This raises the possibility that Nrf1 may regulate the process of TEC pyroptosis in renal IR.

Both intra- and extracellular stimuli are countered by the adaptive immune response of pyroptosis (Shi et al., 2017). NLRP3, which is an inflammatory molecule, activates when it binds with the precursor of caspase-1, procaspase-1, and the adapter protein ASC, followed by conversion of procaspase-1 into catalytic caspase-1. This augments the maturity in addition to the discharge of IL-1β and IL-18, prompting a vigorous inflammatory response (Miao et al., 2010; Xi et al., 2016). To reveal the role of Nrf1 in TEC pyroptosis, we established and verified a stable cell line for Nrf1 overexpression. Nrf1 overexpression reduced the levels of expression of NLRP3, GSDMD-N, and caspase-1. In addition, cell viability and the discharge of LDH, IL-1β, and IL-18 were inhibited, followed by Nrf1 upregulation. These results suggest that Nrf1 overexpression alleviates renal function by inhibiting TEC pyroptosis.

miRNAs participate in a variety of physiological and pathological processes through incomplete pairing with the non-coding region at the 3′UTR of the target gene (Berezikov et al., 2005). Research has shown that miRNA expression is closely associated with TEC pyroptosis in renal IRI. MiR-155 is implicated in renal IRI and regulates TEC pyroptosis (Wu et al., 2016). When miR-506-3p is overexpressed, it curbs pyroptosis of HK-2 cells (Zhu et al., 2020). miR-22-3p inhibits TEC pyroptosis by regulating NLRP3 expression in renal tubular injury (Song et al., 2019). We aimed to understand whether Nrf1 is a target of miRNAs and to assess their effects in the context of TEC pyroptosis in renal IRI. The prediction of target genes using TargetScan, miRDB, and Diana showed that Nrf1 is a target of miR-92a-3p. Moreover, we discovered that miR-92a-3p expression was upregulated in TECs exposed to IRI and was negatively correlated with Nrf1 mRNA expression. Subsequently, it was confirmed by the dual luciferase assay that Nrf1 could be targeted by miR-92a-3p. Thus, we hypothesized that miR-92a-3p could be a novel therapeutic target in renal IRI. We further investigated the role of miR-92a-3p in TEC pyroptosis in renal IRI. The inhibition of miR-92a-3p reduced the levels of NLRP3, caspase1, IL-1β, IL-18, and GSDMD-N expression *in vivo* and *in vitro*. In addition, renal tubule damage and TEC death in IR-induced kidneys were alleviated following miR-92a-3p inhibition. Together, our results show that inhibition of miR-92a-3p alleviates tubular pyroptosis in renal IRI by preventing the genetic suppression of Nrf1. Recently, a clinical study showed that miR-92a in the serum was a potential novel biomarker of rapid aortic valve calcification (Nader et al., 2017), which brought our inspiration. Therefore, the next step of our study is to find out the possible correlation between the expression of miR-92a-3p and the severity and prognosis of ischemic AKI.

The two chief factors responsible for the pathogenesis of renal IRI are oxidative stress and pyroptosis. Oxidative stress causes tubular injury and induces pyroptosis, which leads to oxidative stress, thus creating a vicious cycle that intensifies renal damage (Diao et al., 2019; Zhu et al., 2020). In our study, miR-92a-3p knockdown significantly alleviated oxidative stress in TECs. HO-1 is thought to be a cytoprotective stress-response protein. Under normal conditions, HO-1 expression may or may not be low, while under stress conditions, it can reduce apoptosis, inflammation, and oxidative stress (Lever et al., 2016; Waza et al., 2018). Previous studies have shown that HO-1 alleviates renal damage, oxidative stress, and inflammatory reactions during renal IRI (Salom et al., 2007; Barakat et al., 2018). Our results show that the upregulation of Nrf1 leads to an increased expression of HO-1 in OGD/R-treated TECs and that the inhibition of HO-1 could reduce the protective effect of Nrf1 overexpression on OGD/R-induced TECs from oxidative stress and pyroptosis. This suggests that the inhibited miR-92a-3p diminishes TECs oxidative stress and pyroptosis via HO-1.

In conclusion, miR-92a-3p plays an essential role in regulating TEC pyroptosis by targeting Nrf1 via HO-1.

## Data Availability Statement

The original contributions presented in the study are included in the article/supplementary material, further inquiries can be directed to the corresponding author/s.

## Ethics Statement

The animal study was reviewed and approved by the Institutional Animal Care and Use Committee of Qingdao Municipal Hospital.

## Author Contributions

RW and CX designed the research, analyzed the data, and drafted the manuscript. HZo, YZ, and JD performed the experiments. CX, HZu, QS, and HQ helped with data acquisition and discussion. CX and RW analyzed the data and prepared the figures. All authors contributed to the article and approved the submitted version.

## Conflict of Interest

The authors declare that the research was conducted in the absence of any commercial or financial relationships that could be construed as a potential conflict of interest.

## References

[B1] Al-Bataineh M. M. Kinlough C. L. Poland P. A. Pastor-Soler N. M. Sutton T. A. Mang H. E. (2016). Muc1 enhances the beta-catenin protective pathway during ischemia-reperfusion injury. *Am. J. Physiol. Renal Physiol.* 310 F569–F579. 26739894 10.1152/ajprenal.00520.2015 PMC4796271

[B2] Barakat M. Gabr M. M. Zhran F. El-Adl M. Hussein A. M. Barakat N. (2018). Upregulation of heme oxygenase 1 (HO-1) attenuates kidney damage, oxidative stress and inflammatory reaction during renal ischemia/reperfusion injury. *Gen. Physiol. Biophys.* 37 193–204. 10.4149/gpb_2017018 29593125

[B3] Bastin A. J. Ostermann M. Slack A. J. Diller G. P. Finney S. J. Evans T. W. (2013). Acute kidney injury after cardiac surgery according to Risk/Injury/Failure/Loss/End-stage, acute kidney injury network, and kidney disease: improving global outcomes classifications. *J. Crit. Care* 28 389–396. 10.1016/j.jcrc.2012.12.008 23743540

[B4] Bellomo R. Kellum J. A. Ronco C. (2012). Acute kidney injury. *Lancet* 380 756–766. 22617274 10.1016/S0140-6736(11)61454-2

[B5] Berezikov E. Guryev V. van de Belt J. Wienholds E. Plasterk R. H. Cuppen E. (2005). Phylogenetic shadowing and computational identification of human microRNA genes. *Cell* 120 21–24. 10.1016/j.cell.2004.12.031 15652478

[B6] Chawla L. S. Eggers P. W. Star R. A. Kimmel P. L. (2014). Acute kidney injury and chronic kidney disease as interconnected syndromes. *N. Engl. J. Med.* 371 58–66. 24988558 10.1056/NEJMra1214243 PMC9720902

[B7] Diao C. Chen Z. Qiu T. Liu H. Yang Y. Liu X. (2019). Inhibition of PRMT5 attenuates oxidative stress-induced pyroptosis via activation of the Nrf2/HO-1 signal pathway in a mouse model of renal ischemia-reperfusion injury. *Oxid. Med. Cell. Longev.* 2019:2345658. 10.1155/2019/2345658 PMC6899313 31885778

[B8] Evans M. J. Scarpulla R. C. (1990). NRF-1: a trans-activator of nuclear-encoded respiratory genes in animal cells. *Genes Dev.* 4 1023–1034. 10.1101/gad.4.6.1023 2166701

[B9] Galluzzi L. Vitale I. Aaronson S. A. Abrams J. M. Adam D. Agostinis P. (2018). Molecular mechanisms of cell death: recommendations of the Nomenclature Committee on Cell Death 2018. *Cell Death Differ.* 25 486–541. 29362479 10.1038/s41418-017-0012-4 PMC5864239

[B10] Gill N. Nally J. J. Fatica R. A. (2005). Renal failure secondary to acute tubular necrosis: epidemiology, diagnosis, and management. *Chest* 128 2847–2863. 10.1378/chest.128.4.2847 16236963

[B11] Gyuraszova M. Gurecka R. Babickova J. Tothova L. (2020). Oxidative stress in the pathophysiology of kidney disease: implications for noninvasive monitoring and identification of biomarkers. *Oxid. Med. Cell. Longev.* 2020:5478708. 10.1155/2020/5478708 PMC7007944 32082479

[B12] Ha M. Kim V. N. (2014). Regulation of microRNA biogenesis. *Nat. Rev. Mol. Cell Biol.* 15 509–524. 25027649 10.1038/nrm3838

[B13] Han S. J. Lee H. T. (2019). Mechanisms and therapeutic targets of ischemic acute kidney injury. *Kidney Res. Clin. Pract.* 38 427–440. 10.23876/j.krcp.19.062 31537053 PMC6913588

[B14] Hou Y. Liu Z. Zuo Z. Gao T. Fu J. Wang H. (2018). Adipocyte-specific deficiency of Nfe2l1 disrupts plasticity of white adipose tissues and metabolic homeostasis in mice. *Biochem. Biophys. Res. Commun.* 503 264–270. 10.1016/j.bbrc.2018.06.013 29935181

[B15] Hsieh P. F. Liu S. F. Hung T. J. Hung C. Y. Liu G. Z. Chuang L. Y. (2016). Elucidation of the therapeutic role of mitochondrial biogenesis transducers NRF-1 in the regulation of renal fibrosis. *Exp. Cell Res.* 349 23–31. 10.1016/j.yexcr.2016.09.005 27634749

[B16] Huang H. Ni H. Ma K. Zou J. (2019). ANGPTL2 regulates autophagy through the MEK/ERK/Nrf-1 pathway and affects the progression of renal fibrosis in diabetic nephropathy. *Am. J. Transl. Res.* 11 5472–5486. 31632523 PMC6789235

[B17] Jiang D. Fu C. Xiao J. Zhang Z. Zou J. Ye Z. (2019). SGK1 attenuates oxidative stress-induced renal tubular epithelial cell injury by regulating mitochondrial function. *Oxid. Med. Cell. Longev.* 2019:2013594. 10.1155/2019/2013594 PMC6766675 31641423

[B18] Kovacs S. B. Miao E. A. (2017). Gasdermins: effectors of pyroptosis. *Trends Cell Biol.* 27 673–684. 10.1016/j.tcb.2017.05.005 28619472 PMC5565696

[B19] Lever J. M. Boddu R. George J. F. Agarwal A. (2016). Heme oxygenase-1 in kidney health and disease. *Antioxid. Redox Signal.* 25 165–183. 10.1089/ars.2016.6659 26906116 PMC4948210

[B20] Lin F. (2017). Autophagy in renal tubular injury and repair. *Acta Physiol. (Oxf.)* 220 229–237. 10.1111/apha.12852 28112877 PMC7814874

[B21] Linkermann A. Chen G. Dong G. Kunzendorf U. Krautwald S. Dong Z. (2014). Regulated cell death in AKI. *J. Am. Soc. Nephrol.* 25 2689–2701. 10.1681/asn.2014030262 24925726 PMC4243360

[B22] Liu H. Chen Z. Weng X. Chen H. Du Y. Diao C. (2020). Enhancer of zeste homolog 2 modulates oxidative stress-mediated pyroptosis in vitro and in a mouse kidney ischemia-reperfusion injury model. *FASEB J.* 34 835–852. 10.1096/fj.201901816r 31914694

[B23] Livak K. J. Schmittgen T. D. (2001). Analysis of relative gene expression data using real-time quantitative PCR and the 2(-Delta Delta C(T)) method. *Methods* 25 402–408. 10.1006/meth.2001.1262 11846609

[B24] Miao E. A. Leaf I. A. Treuting P. M. Mao D. P. Dors M. Sarkar A. (2010). Caspase-1-induced pyroptosis is an innate immune effector mechanism against intracellular bacteria. *Nat. Immunol.* 11 1136–1142. 10.1038/ni.1960 21057511 PMC3058225

[B25] Nader J. Metzinger-Le M. V. Maitrias P. Humbert J. R. Brigant B. Tribouilloy C. (2017). miR-92a: a novel potential biomarker of rapid aortic valve calcification. *J. Heart Valve Dis.* 26 327–333. 29092119

[B26] Noh M. R. Kim J. I. Han S. J. Lee T. J. Park K. M. (2015). C/EBP homologous protein (CHOP) gene deficiency attenuates renal ischemia/reperfusion injury in mice. *Biochim. Biophys. Acta* 1852 1895–1901. 10.1016/j.bbadis.2015.06.004 26071644

[B27] Perico N. Remuzzi G. (2015). Acute kidney injury: more awareness needed, globally. *Lancet* 386 1425–1427. 10.1016/s0140-6736(15)00425-0 26466028

[B28] Ravikumar P. Li L. Ye J. Shi M. Taniguchi M. Zhang J. (2016). alphaKlotho deficiency in acute kidney injury contributes to lung damage. *J. Appl. Physiol. (1985)* 120 723–732. 10.1152/japplphysiol.00792.2015 26718784 PMC4824041

[B29] Salom M. G. Ceron S. N. Rodriguez F. Lopez B. Hernandez I. Martinez J. G. (2007). Heme oxygenase-1 induction improves ischemic renal failure: role of nitric oxide and peroxynitrite. *Am. J. Physiol. Heart Circ. Physiol.* 293 H3542–H3549. 17890422 10.1152/ajpheart.00977.2007

[B30] Scarpulla R. C. (1996). Nuclear respiratory factors and the pathways of nuclear-mitochondrial interaction. *Trends Cardiovasc. Med.* 6 39–45. 10.1016/1050-1738(95)00129-8 21232273

[B31] Shi J. Gao W. Shao F. (2017). Pyroptosis: gasdermin-mediated programmed necrotic cell death. *Trends Biochem. Sci.* 42 245–254. 10.1016/j.tibs.2016.10.004 27932073

[B32] Shi J. Zhao Y. Wang K. Shi X. Wang Y. Huang H. (2015). Cleavage of GSDMD by inflammatory caspases determines pyroptotic cell death. *Nature* 526 660–665. 10.1038/nature15514 26375003

[B33] Song Z. Zhang Y. Gong B. Xu H. Hao Z. Liang C. (2019). Long noncoding RNA LINC00339 promotes renal tubular epithelial pyroptosis by regulating the miR-22-3p/NLRP3 axis in calcium oxalate-induced kidney stone. *J. Cell. Biochem.* 120 10452–10462. 10.1002/jcb.28330 30614043

[B34] Tajima T. Yoshifuji A. Matsui A. Itoh T. Uchiyama K. Kanda T. (2019). beta-hydroxybutyrate attenuates renal ischemia-reperfusion injury through its anti-pyroptotic effects. *Kidney Int.* 95 1120–1137. 10.1016/j.kint.2018.11.034 30826015

[B35] Waza A. A. Hamid Z. Ali S. Bhat S. A. Bhat M. A. (2018). A review on heme oxygenase-1 induction: is it a necessary evil. *Inflamm. Res.* 67 579–588. 10.1007/s00011-018-1151-x 29693710

[B36] Wu H. Huang T. Ying L. Han C. Li D. Xu Y. (2016). MiR-155 is involved in renal ischemia-reperfusion injury via direct targeting of foxo3a and regulating renal tubular cell pyroptosis. *Cell. Physiol. Biochem.* 40 1692–1705. 10.1159/000453218 28006785

[B37] Xi H. Zhang Y. Xu Y. Yang W. Y. Jiang X. Sha X. (2016). Caspase-1 inflammasome activation mediates homocysteine-induced pyrop-apoptosis in endothelial cells. *Circ. Res.* 118 1525–1539. 10.1161/circresaha.116.308501 27006445 PMC4867131

[B38] Xiao C. Zhao H. Zhu H. Zhang Y. Su Q. Zhao F. (2020). Tisp40 induces tubular epithelial cell GSDMD-mediated pyroptosis in renal ischemia-reperfusion injury via NF-kappaB signaling. *Front. Physiol.* 11:906. 10.3389/fphys.2020.00906 32903383 PMC7438479

[B39] Xiao C. C. Zhang J. Luo P. C. Qin C. Du Y. Ning J. Z. (2017). Identification of Tisp40 as an essential regulator of renal tubulointerstitial fibrosis via TGF-beta/smads pathway. *Cell. Physiol. Biochem.* 42 697–712. 28618421 10.1159/000477887

[B40] Zhang Y. Manning B. D. (2015). mTORC1 signaling activates NRF1 to increase cellular proteasome levels. *Cell Cycle* 14 2011–2017. 26017155 10.1080/15384101.2015.1044188 PMC4613906

[B41] Zhu B. Cheng X. Jiang Y. Cheng M. Chen L. Bao J. (2020). Silencing of KCNQ1OT1 decreases oxidative stress and pyroptosis of renal tubular epithelial cells. *Diabetes Metab. Syndr. Obes.* 13 365–375. 32104033 10.2147/DMSO.S225791 PMC7025682

