# Corrigendum: Identification of microRNA-92a-3p as an essential regulator of tubular epithelial cell pyroptosis by targeting Nrf1 via HO-1

**DOI:** 10.3389/fgene.2023.1272660

**Published:** 2023-11-01

**Authors:** Renhe Wang, Haijun Zhao, Yingyu Zhang, Hai Zhu, Qiuju Su, Haiyan Qi, Jun Deng, Chengcheng Xiao

**Affiliations:** ^1^ Department of Traditional Chinese Medicine, Qingdao Municipal Hospital, Qingdao University, Qingdao, China; ^2^ Department of Urology, Qingdao Municipal Hospital, Qingdao University, Qingdao, China

**Keywords:** renal ischemia–reperfusion injury, miR-92a-3p, Nrf1, pyroptosis, tubular epithelial cell, HO-1

In the published article, there was an error in Figure 5A as published. A visual field of IL-18 staining in Sham antagomir NC group was misplaced in the Sham antagomir miR-92a-3p group, when assembled. The corrected Figure 5 is given below.

**FIGURE 5:**
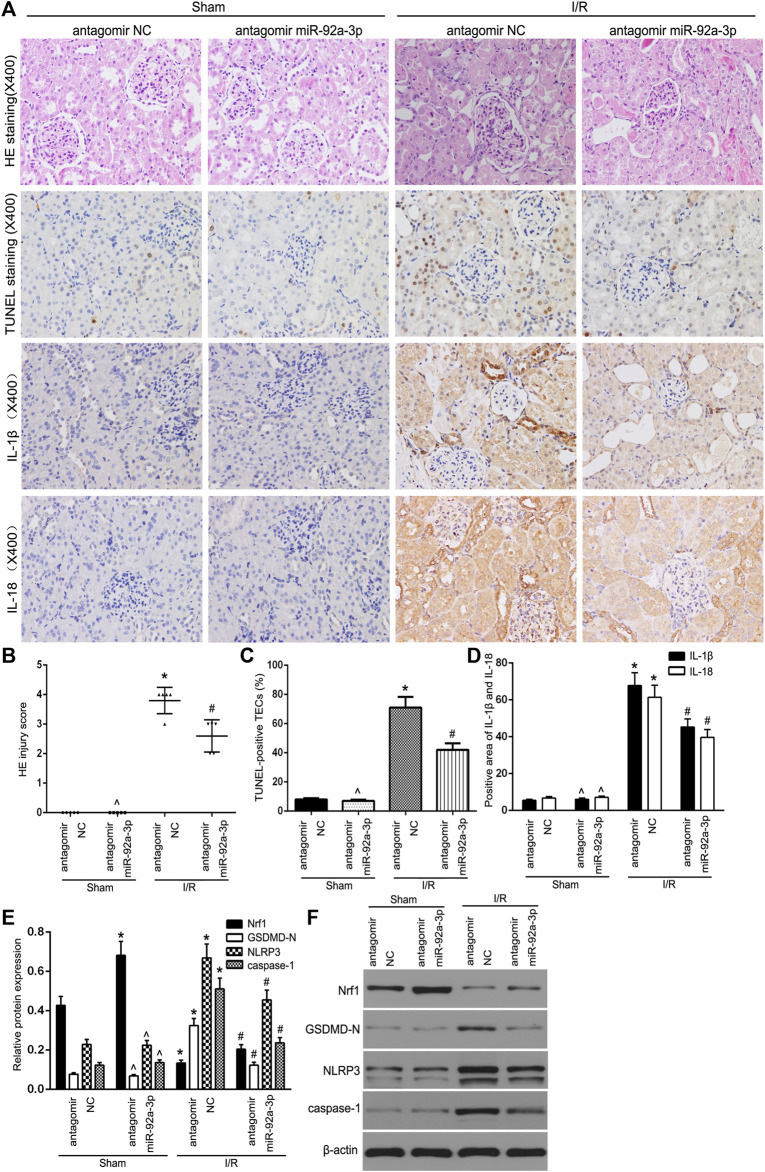
The inhibition of miR-92a-3p alleviated TEC pyroptosis in I/R-induced kidney of mice. **(A)** Representative photomicrographs of tubular cell injury in mouse kidney tissue sections with HE staining, TUNEL staining, and representative photomicrographs of IL-1β and IL-18 expression in mouse kidney tissue sections by immunohistochemistry, 400×, scale bar = 20 µm. **(B)** Statistical quantification analysis showed the injury score of HE staining in the kidney tissues. **(C)** Statistical analysis showed the percentage of TUNEL-positive TECs in the kidney tissues. **(D)** Statistical analysis showed the positive area of IL-1β and IL-18 in the kidney tissues. **(E,F)** Western blot analysis of Nrf1, GSDMD-N, NLRP3, and caspase-1 expression in mouse kidney tissue sections. SCr levels **(G)** and BUN levels **(H)** were detected in mice. Data are expressed as the mean ± SD. *n* = 5 per group. *P < 0.05 vs. antagomir NC sham group. ∧*P* > 0.05 vs. antagomir NC sham group. #*P* < 0.05 vs. I/R-induced antagomir NC group, one-way ANOVA.

The authors apologize for this error and state that this does not change the scientific conclusions of the article in any way. The original article has been updated.

